# The Foreign Journals: Surgery

**Published:** 1842-07

**Authors:** 


### SURGERY.

### *Cure of Traumatic Tetanus by Division of the Nerve supplying the wounded part.* By Professor Pecchioli, of Siena.

Two cases are related in which this method was successfully employed. The first patient was a lad 16 years old, who had received a lacerated wound of the great toe of the left foot, and in whom after severe traumatic fever the signs of tetanus set in on the third day from the reception of the injury, the wound at the same time being highly inflamed, and the whole foot painful. The branch of the internal saphenus nerve going to the toe was divided a few hours after the tetanic symptoms were established; the pain in the wound immediately ceased, the convulsions became less and gradually disappeared, the fever continued only two days longer, and the patient was ultimately discharged quite well. The second case was that of a man 30 years old, who had received a wound from an axe across the left foot, and in whom signs of tetanus appeared on the fourth day. He, as is usual in Italy, had been profusely bled for the fever that followed the injury to his foot. On the day after the tetanic symptoms were completely established, the anterior tibial nerve was divided an inch above the wound, which was acutely inflamed. The symptoms subsided more slowly than in the former case, but they at last ceased, and he also was discharged cured.

*Bullettino delle Scienze Mediche. Marzo,* 1841.

---

### *On Fractures of the Lower Extremity of the Radius.* By M. Velpeau.

M. Velpeau entirely contradicts Dupuytren's account of the ill consequences of this injury when treated as a sprain or a dislocation, and says, that while he has seen many cases, which thus treated, have recovered with scarcely any deformity and no loss of motion, he has known many more which, though treated carefully with approved apparatus, have presented all the bad results of stiffness of the joint and defective power of the muscles. He believes that all the apparatus hitherto described do more harm than good; and says, the only useful mode of treatment is that with the dextrine bandage. After reducing the fracture he puts a compress, wet with camphorated spirit, round the wrist, and applies a dry bandage very lightly from the roots of the fingers to the middle of the arm. Over this he places graduated compresses reaching to the beginning of the metacarpus, and then an anterior and posterior splint of moistened pasteboard, which are moulded exactly on the parts they have to cover, and descend to the roots of the fingers. A bandage wet with starch is then rolled in a double layer, from the fingers to a short distance above the elbow; and, till it has dried, all the parts are kept in their places by two long wooden splints. These last, however, are removed after six and eight hours, and the part left in its immoveable bandage supported in a sling, for from twenty to thirty days, by which time the union of the fracture is generally perfected. There are but few cases, M. Velpeau adds, in which this method of treatment is not sufficient.

*Gazette des Hôpitaux. Janvier* 13, 1842.

---

*Dislocation of the Thumb.* By M. RADAT.

THIS was a case of dislocation of the last phalanx of the thumb backwards. A soldier was running on a moist clay soil, when he slipt, and putting out his hand to save himself, his thumb stuck in the stiff ground, and, as it was thus fixed, he fell over it. The symptoms were, considerable shortening of the thumb, retroversion of the phalanx in the direction of extension, so that it formed almost a right angle with the metacarpal bone, immobility of the joint, and a prominence on the palmar surface. It was reduced in the following manner: A piece of stout bandage was taken with a hole in its middle, through which the thumb was passed; the two ends being then carried forwards, were strapped close down upon the dislocated phalanx, and extension made by pulling them while another person held the metacarpal bone, and a third pressed in the phalanx as soon as it was drawn nearly to the level of the joint.

*Gazette Médicale. Mars* 12, 1842.

---

*Artificial Anus. Closure of the Intestinal Canal by a Tumour in the left Iliac Fossa.* By M. VIDAL (de Poitiers).

THIS case contains an account of the fifth operation performed by M. Amussat for the formation of a false anus. After frequent postponements of the operation because the intestinal canal did not seem to be completely closed, though the abdomen was tender and excessively distended, it was performed on the 10th day, after nothing but gas had been passed by the anus, and on the 40th from the commencement of the obstruction. The ascending colon was with some difficulty opened, and the patient from that time slowly recovered his health, though the tumour which was supposed to be carcinomatous, remained. Ten weeks after the operation he was in a satisfactory state; he had gained much of his former strength; the artificial anus gave free passage to the fæces, and the discharge of these had become less frequent.

*Gazette des Hopitaux. Mars* 26, 1842.

---

*Reunion of two Fingers completely separated from the Hand and each divided into two pieces.* By SIGNOR DELLA FANTERIA.

A GIRL, fourteen years old, was engaged with another person in some domestic occupation, when the latter accidentally let fall a knife which cut off two of her fingers below the first phalanx. The author being soon after summoned found the two pieces in some meal on which the patient's hand was resting at the time of the accident; but he discovered, to his great surprise, that each of them was divided into two portions. However, he determined to try to unite them, and having put the bits together, he kept them all in their places with sutures and strips of plaster. In a few days the adhesion was completed, and the patient ultimately recovered the entire use of her fingers.

It is necessary to mention that the authenticity of this strange case was confirmed by Professors Centofanti and Vacca.

*Annali Universali di Medicina,* 1841; *and Gazette Médicale, Mars* 5, 1842.

### *On the Nature and Treatment of Erysipelas.* By M. Velpeau.

The main object of this paper is to recommend the use of sulphate of protoxide of iron as a local application in cases of simple erysipelas. After the failure of every other means tried in numerous cases, this was tried on forty patients, and in no case did an inflamed spot resist its influence for more than from twenty-four to forty-eight hours. Erratic erysipelas, however, often continued its course.

The modes of applying the remedy are two, namely, in a lotion of thirty grains to the pint of water, kept constantly applied on rags to the inflamed part; and in an ointment composed of one part of the salt in an impalpable powder with four parts of lard, and applied freely three times a day. The former is the more efficacious plan: the only objection to it is that it spoils the rags.

*Annales de la Chirurgie. Fevrier,* 1842.

---

### *On External Fistulæ of the Larynx.* By M. Trousseau.

The cases here detailed are the results of inflammation of the submucous tissue of the larynx producing first ossification, and then necrosis of the cartilages. In some cases the matter which is formed makes its way to the interior of the larynx, and being discharged as fast as it is formed the disease goes steadily on till the necrosed portion is separated, and a cicatrix formed over its place. In others, however, matter forms on the outside of the larynx, and opening in the neck a fistula is produced, which either remains permanently open, or closes for a time and then reopens, and so on. Four striking cases unconnected with phthisis are related, and the main practical point deduced from them is, that when there is an external fistula with necrosis no attempts should be made to close it; for the necrosis is never superficial, but extends through the whole thickness of the ossified cartilage, and if the matter be prevented from discharging itself externally it will certainly continue to be formed, and produce much more serious mischief by pressing inwards upon the mucous membrane of the larynx, or by exciting inflammatory swelling about the glottis.

*Journal des Connaissances, Méd. Chir. Mars,* 1842.

---

### *Radical Cure of Reducible Inguinal Hernia.* By Dr. C. Haller.

Seven cases are given. The operation, as detailed in the first of these, was as follows: The patient, a man of twenty-three years, had ruptured himself in 1837. In 1840, when the operation was performed, his hernia, which was on the right side, had attained the size of a hen's egg, was soft, elastic, and contained a small knuckle of intestine. It made its appearance on the patient coughing or violently exerting himself, but receded on his assuming the horizontal position. The inguinal ring was so far enlarged as to admit the index-finger. The rectum having been emptied by a clyster, the scrotal integument was invaginated in the inguinal canal, by the index-finger of the operator; while, with the right hand, by means of the *sonde à dard* two stitches were made; the one as high as possible on the inner crus of Poupart's ligament; the other just over the angle of the two crura. A ball or cork of lint was introduced into the loop thus formed on the thread; traction was applied to one end of the thread, by which the lint-ball was carried as far as possible up the inguinal canal; the double threads were then separated, and tied down over two quills united by sticking plaster. Ice was applied to the seat of operation; the testicles were supported; the patient was ordered to bed; and put on low diet. There were slight pain and redness, but considerable swelling and hardness around the cylindrical aperture; and on the sixth day there were light febrile symptoms. As suppuration commenced from the punctures, the threads were loosened; the lint slightly withdrawn, and thus a free exit permitted to the matter. Warm applications to the part, daily ablution, and syringing of the invaginated integument, laxative clysters, constituted the treatment. The suppuration continued during fourteen days, and as it declined, the integument gradually subsided into the inguinal canal. On the 28th day from the operation, the patient, with the aid of an elastic band, could stand up, and move about a little. The inguinal canal was appreciably narrowed, and even violent coughing or straining did not reproduce the hernia. In nine months the patient dispensed with the truss. In two or three of the other cases the cure was only temporary, the hernia again descending and carrying the integument before it. But in several instances the operation has been successful.

*Oesterr. Medicinische Jährbucher. März,* 1842.

---

*On the Cure of Spina Bifida.* By M. BEYNARD.

THE patient was a day old, and had a tumour from spina bifida over the third lumbar vertebra. It was as large as a hen's egg, had a narrow base of attachment, and the skin over it was healthy. M. Beynard took two quills, through each of which a string was passed, and placed one on each side of the pedicle of the tumour, which he then constricted by gradually tightening the string till the skin became hard and rather deeply coloured. The quills were retained in their places by strips of plaster, and on the following day the tumour was livid, hard, cold, and covered with vesicles. Some fluid was let out of it till it became flaccid, and its base was more tightly constricted. On the 7th day, the pedicle being dry and hard was cut across, and the wound thus made was simply strapped: it had no communication with the spinal canal, the walls of the neck of the sac having been completely obliterated by the pressure. On the 26th day a cicatrix had formed and the cure was perfected.

*Bulletin Médical de Bordeaux. Fevr.* 1842

---

*On Gilding of Surgical Instruments.* By M. CHARRIERE.

THE electrotype has been applied to this purpose in Paris. A letter from M. Charriere was read at the Institute on the 21st of March, in which he says, "Having gilded by M. de Rustz's process a considerable number of surgical instruments and pieces of cutlery, I have submitted them to experiments which seem to me to merit attention. The cutting instruments which I have repeatedly tested on the dead body, have suffered no damage either in the quality of their edge or in their gilding; and the instruments for pressing have preserved all their power of resistance. I have moreover obtained a positive proof that the instruments thus gilded are not subject to rust; and this is an advantage of which the importance may be easily understood, especially for instruments which are intended to remain for some time in the body. I may add that the silver and the platinum plating, applied in the same manner, afford the same results as the gilding."

*Gazette des Hôpitaux. Mars* 24, 1842.

---

*Case of Exophthalmus from Atheroma of the Orbit.* By Professor ROSAS.

A MAN, aged forty-five, received in 1827 a contusion on the root of the nose; and in June 1830, a sharp blow on the inner angle of the right eye. These accidents were followed by swelling, &c. This swelling went on increasing, advancing in a direction from without inwards to the root of the nose, and pressing at the same time the eye out of the orbit and downwards. This organ was, however, neither inflamed nor swollen. Professor Rosas saw the patient for the first time in 1831; the following were the appearances presented. (It may here be noticed that prior to this, the man had been advised by several practitioners to submit to the extirpation of the eye.) The eye (as is seen in an annexed engraving) was situated low on cheek, yet not much altered either in its general figure or interior appearance and structure: but vision was entirely destroyed, and even the sensation of light extremely imperfect. There was not much suffering; but the general health of the patient was affected. Obscure fluctuation in the site of the swelling, and the continuance of the lachrymal secretion, &c. guided Professor Rosas in his diagnosis, and led him to believe that the swelling was not owing to exostosis, or enlargement of the lachrymal gland. The operation was simple. An eye-bistoury was introduced about the middle part of the upper eyebrow, and cautiously pushed into the orbit, until resistance was experienced. Immediately there issued a dirty greenish-gray gelatinous fluid, of the consistence of syrup. This discharge continued to the extent of six ounces, and gradually as it proceeded, the eyeball receded into the orbit. Pressure with the finger was then exercised to complete the evacuation of the matter: and forthwith the patient recovered not merely an undoubted perception of light, but also the power of distinguishing the outline of near objects. The cavity was then syringed and filled with lint; cold water applications for twelve hours, purgatives, and restricted diet prescribed. The case proceeded satisfactorily, and a perfect cure was effected, though a slight fistulous discharge seems to have taken place from the place of incision for five months. This closed ultimately, and vision was perfectly restored.

*Oesterreich. Medicinische Wochenschrift. Jänuar* 1, 1842.

---

*On the operation for Hare-lip.* By LOUIS BERG-DE-VARSOVIC.

THE author says that in all the operations for hare-lip there is no prevention of the deformity subsequently arising from the existence of a notch in the upper lip at the lower end of the cicatrix. To prevent this he recommends that the edges of the fissure should be cut off, not in a straight line, but with an angle of which the shorter leg is directed upwards and outwards, and the longer at nearly a right angle to the former, upwards and inwards to the upper part of the fissure. By this mode of incision he saves an angle of the lip on each side at the lowest part of the fissure; and these when turned downwards so that their cut surfaces may be opposed will, if they are well proportioned, exactly fill the space where the notch usually exists. According to the direction, the position, and the extent of the fissure, the direction and extent of the portions excised must vary; but the same principle is applicable to all cases. The operation is performed with a sharp-pointed narrow scalpel.

*Journal de Médecine et d'Histoire Naturelle, par l'Académie Méd.-Chir. de St. Petersbourg*, 1841.

---

*On Hemorrhage after Lithotomy.* By M. BEGIN.

THE author says one patient in every five or six that are cut for stone dies: and about a fourth of these die from hemorrhage, the source of which is scarcely ever to be ascertained, but which probably often proceeds from the small vessels unnaturally dilated by the irritation of the calculus. He recommends irrigation of the perineum with cold water in such cases. A flexible tube being dipped into a reservoir placed above the patient, who lies on his side with his buttocks turned over the side of the bed, some one should raise his upper buttock and direct the stream of water continually upon the perineum and the wound, and let it pass off the bed upon a sheet of oiled silk. He relates several cases of severe hemorrhages successfully treated in this manner.

*Bulletin de l'Acad. Roy. de Médecine. Mars,* 1842.

---

*On the Radical Cure (so called) of Reducible Hernia.*
By Dr. C. L. SIGMUND, of Vienna.

THE author says that he is acquainted with several cases in which operations have been performed after the method of Gèrdy, Signoroni, and others, for the cure of hernia, and that he has himself been more than once induced to operate; but he has written this paper to prove that there are scarcely any, if indeed there be any, cases in which such a proceeding should be adopted. In some of the cases which he has witnessed the patients have only preserved their lives after the greatest peril; in a few death has been the result; in all who have recovered the cure has been but temporary, and the hernia has again protruded and required the wearing of a truss. The chief dangers of the operation, in whatever manner it is performed, are peritonitis, which is not avoidable by any circumspection, and inflammation of the aponeurosis of the abdominal muscles, with suppuration spreading along them. If these do not occur, the inflammation in the inguinal canal is scarcely ever sufficient to effect a firm union of the walls of the sac, and on the least exertion the intestine is forced through the adhesions. The author concludes, therefore, that this operation must again be excluded from the practice of surgery, except in cases where its performance is urged by patients in whose cases there is no obvious reason to believe that it will be injurious.

*Hufeland's Journal der praktischen Heilkunde. März,* 1841.

---

*Case of Varicose Tumour situated in the Groin simulating a strangulated Crural Hernia in an individual who died of an acute Entero-peritonitis, produced by a Cancer of the Rectum.* By Dr. DE CASTELLA.

THE long title of this paper tells us all that is interesting in it. The chance of having committed an important error by supposing that the patient had a strangulated hernia was not discovered till after death, for though the author had examined the inguinal region he had not observed the swelling. Probably also a sufficient means of diagnosis would have been the absence of signs of peritonitis at the tumour while they existed over the rest of the abdomen.

*Gazette Médicale. Mars* 12, 1842.

